# Investigation of LRTOMT gene (locus DFNB63) mutations in Iranian patients with autosomal recessive non-syndromic hearing loss

**Published:** 2013

**Authors:** Seyyed Hossein Taghizadeh, Seyyed Reza Kazeminezhad, Seyyed Ali Asghar Sefidgar, Nasrin Yazdanpanahi, Mohammad Amin Tabatabaeifar, Ahmad Yousefi, Seyyed Mohammad Lesani, Marziyeh Abolhasani, Morteza Hashemzadeh Chaleshtori

**Affiliations:** 1 *Department of Genetic, Faculty of Science, ShahidChamran University of Ahwaz, Ahwaz, Iran.*; 2 *Cellular and Molecular Biology Research Center (CMBRC), Babol University of Medical Sciences, Babol, Iran.*; 3 *Department of Biochemistry and Genetics, Falavarjan Branch, Islamic Azad University, Isfahan, Iran.*; 4 * Department of Genetics, Faculty of Medicine, JundiShapur University of Medical Sciences, Ahwaz, Iran.*; 5 *Department of Basic Sciences, Faculty of Veterinary Medicine, Shahrekord University, Shahrekord, Iran.*; 6 *Department of Genetics, Faculty of Science, Shahrekord University, Shahrekord, Iran.*; 7 *Cellular and Molecular Research Center, Shahrekord University of Medical Sciences, Shahrekord, Iran.*

**Keywords:** Non - syndromic sporadic hearing loss, DFNB63, LRTOMT gene, PCR - SSCP, heteroduplex, Iran

## Abstract

Hearing loss (HL) is the most frequent sensory defect affecting 1 in 1000 neonates. This can occur due to genetic or environmental causes or both. The genetic causes are very heterogenous and over 100 loci have been identified to cause autosomal recessive non - syndromic hearing loss (ARNSHL). The aim of this study was to determine the contribution of the LRTOMT gene mutations in causing ARNSHL. One hundred fifty seven pupils affected with ARNSHL from Azarbaijan Sharghi, Kordestan, Gilan and Golestan provinces, north and west of Iran, were ascertained. In this descriptive - laboratory study, the presence of *LRTOMT* mutations were initially checked using PCR – Single - strand conformation polymorphism (SSCP) and heteroduplex analysis (HA) strategy. Samples with shifted bands on the gel were confirmed by DNA sequencing method. The PCR-SSCP/HA and the subsequent direct DNA sequencing showed no mutation in the population studied. We conclude that *LRTOMT* mutations have no role in causing sporadic deafness in the studied population. Further studies on other populations and samples could clarify the exact role of *LRTOMT* mutations.

Hearing loss (HL) is the most common sensory defect in human. One infant out of 1000 has pre - lingual HL. Some degrees of HL affect 4% of the people under 45 and 10% of 65 or above (-). HL has a wide spectrum of clinical manifestations: congenital or late onset, conductional or sensory - nervous, syndromic or non - syndromic    (4). The causes of HL can be genetical, environmental or both. More than 60% of the cases are assumed to be genetic. Over 70% of the genetic HL, are non - syndromic in which HL is the only evident manifestation. Almost 80% of the latter are autosomal recessive (1, 3-4). The phenotype is generally more severe in autosomal recessive non - syndromic HL (ARNSHL) and is usually manifested in pre-lingual cases    (4, 5).

The estimations show that 1% of the human genes are involved in the hearing process. More than 100 loci have been identified in NSHL. Thus, HL could be regarded as one of the most heterogeneous traits in human (2).

Thus far, few studies have been performed on the sporadic form of NSHL in Iran and most of the investigation have been limited to a specific locus, DFNB1 (connexin-26 gene). Genetic studies of other loci are necessary to identify the causes and possible mechanisms of this disease. The results of these studies can definitely help to screen this type of HL and perform a more suitable genetic counseling.

To date, few investigations have been carried out about LRTOMT mutations in Iran and frequency of these mutations is not exactly known among Iranian ARNSHL patients (6). LRTOMT gene consists of 10 exons with 2 alternate reading frames and encodes two different proteins: LRTOMT 1 and LRTOMT 2 which are different in starting the translating codons. Exons 5, 7 and 8 have double reading frame. LRTOMT 1 has a transmembrane protein enriched of leucine whose function is unclear. LRTOMT 2 is an O - methyl transferase. The defects in O – methyl transferase protein have been noted to cause NSHL (7).

The current study was carried out on HL patients recruited from different provinces of Iran to screen exons 1, 2, 3, 5 and 8 of the *LRTOMT* gene (DFNB 63 locus) for mutations.

## Materials and Methods

**Sampling**

One hundred fifty seven unrelated Iranian ARNSHL pupils from 13 elementary schools with average age of 11 years from four provinces of north and west of Iran (Azarbaijan Sharghi, Kordestan, Gilan and Golestan provinces) were included in this descriptive – laboratory study in 2011. About 5 ml peripheral blood sample from each individual was collected in tubes containing EDTA (0.5 ml). The samples were kept in - 20ºC until use. A questionnaire was filled out for any family detailing clinical and demographical information and drawing family pedigree. All patients had mild to severe HL, were negative for GJB 2 mutations in our previous study and had not syndromic deafness, inflammatory middle ear disease or specific environmental ethiology according to examination performed by audiologist and general physician (8).

The study was approved by the Review Board of Shahrekord University of Medical Sciences.

**DNA extraction**

Salting out and phenol-chloroform methods were exploited for DNA extraction.

**PCR:** DNA samples were amplified using PCR method. Primers were designed and then blasted using the Gene Runner software (Hastings Software Inc, Hastings, NY). For exon 5, two sets of overlapping primers were designed due to its lengthy size (Table1). Site Directed Mutagenesis method by PCR, using primers with changes in one nucleotide, was used to make positive control samples. Standard PCR optimization was applied separately for each amplicon in 25 µl volume reactions in a thermal cycler machine.

**SSCP and HA**

Microtubes containing PCR products were put in 96ºC for 15 minutes. The samples were transferred on the ice immediately and kept in ice before loading on the *polyacrylamide gel***.** TBE buffer, 1x was used in the gel and TBE 0.6 x was used in the tank (Table 2). Both mutated samples and hetero-duplex analysis were used toincrease the sensitivity of SSCP. In order to perform hetero-duplex analysis, 3 ml EDTA (0.5M) was added to 2 ml PCR products for each sample. Bands were visualized by standard silver staining (9).

**Sequencing of PCR products**

The suspected samples with shifts on the SSCP gel and hetero-duplex were subjected to direct DNA sequencing of exons 1, 2, 3, 5 and 8 of the LRTOMT gene in an ABI 3730XL automated sequencer (Applied Biosystems) (Macrogen, South Korea) for the final verification.

## Results

A total of 157 deaf students, consisting of 85 males and 72 females age ranges from 9 to 13, from families with a history of ARNSHL, who suffered from mild to profound sensory neural HL with no other clinical findings were recruited. After applying quality control measures, no shifts were seen for the tested exons. 

**Table 1:** PCR pieces size and primers sequences used in this study

**Primer sequence** **(3→5)**	**Primer Type**	**Pieces size (bp)**	**Exon**
GAAAGCAGTTGCCATGGAGT GTGGGGGAAATCTCAGATCC	F R	196	E1
CAGCTTTACTTTAGCTAACAAATTGGA AGCCCCATTACCTTTCCATC	F R	290	E2
GCAAGGATGCAAGGAAGAGT GCTCCTGTACCGAAGTGTTC GCTCCTGTACCGAAGGGTTC	F R *RM	248	E3a
GCACCTTTGAATGATGGCCT ACTCTCCCCTACCCTCCAAA GCACCTTTGAATGATTGCCT	F R FM*	259	E3b
AGTATGGCTGTGGAGGGTTG TTCCTCCATGGGGTTCCCAT TTCCTCCATGGGGTTACCAT	F R RM*	270	E5a
GTGAATAAGCTGGCTGTCCT CAAGGAATGGAGGGACTTGA GTGAATAAGCTGGCGGTCCT	F R FM*	277	E5b
AGGATAATAATTGCTACTGGCAAAA GTGAGCACGTAGCTGAAG GTGAGCACGTAGCGGAAG	F R RM*	264	E8a
GGCACTACTTCCGATTGCTG ATCCCAAATATTCCTTCACTGTCTT GGCACTACTTCCGATGGCTG	F R FM*	262	E8b

M*: Mutant primers

**Table 2:** SSCP condition for exons of *LRTOMT* gene

**Temperature(** ^o^ **C)**	**Voltage(V)**	**MA**	**Time(h)**	**Gel ** **den** **sity(%)**	**Exon**
10	280	32	6.5	8	Exon1
10	200	27	14	10	Exon2
10	300	31	7	8	Exon3a
10	200	29	6.5	8	Exon3b
10	200	32	7.5	8	Exon5a
10	200	31	7.5	8	Exon5b
10	280	32	7.5	8	Exon8a
10	260	30	8	8	Exon8b

## Discussion

As all subjects in this study had not GJB2 mutations, the most prevalent deafness responsible gene, in our previous study (8) and the frequency of LRTOMT mutations is not known exactly among Iranian deaf individuals, we decided to investigate these mutations among deaf students from Azerbaijan Sharghi, Kordestan, Gilan and Golestan provinces, north and west of Iran.

Exons 1-5 and 8, including hot spot exons 2, 3, 8 of LRTOMT were selected for mutation analysis. The results showed the lack of mutation in the 157 studied samples negative for Connexin-26 gene mutations.

Overall, few studies have been done on this gene: Tlili et al. identified the DFNB63 locus on 11q13.3-q13.4 chromosome related to deafness    (1). Ahmed et al. identified the gene and the encoded protein structure. They reported 4 different homozygous mutations in *LRTOMT* gene (7). Finally, Du et al. analyzed the *LRTOMT* gene in 192 deaf children and identified homozygosity for a nonsense mutation in one family (11). On a series of Iranian autosomal recessive NSHL samples, a family was found to be affected with a 1 bp deletion (a frameshift mutation) (11). In the current study, the applied method was PCR-SSCP accompanied by HA, which was a simple, cheap and sensitive method (9). Using this combination, the sensitivity will increase considerably. In this study, all the variations introduced by site directed mutagenesis were recognized using the method.

Although other coding regions of the gene as well as the promoter of* LRTOMT* which were not studied in the current investigation, may be mutated, our findings suggest that the gene might play no considerable role in causing ARNSHL. However, further research is warranted for exact conclusion. Other exons and intronic sequences of LRTOMT should be investigated to clarify the role of this gene in causing deafness in Iranians.
